# Low-Cost High-Gain Transmitarray with Beam-Scanning Enhancement Based on Hybrid Phase Distribution Method

**DOI:** 10.3390/s26092721

**Published:** 2026-04-28

**Authors:** Ming Wu, Hang Ren, Jinyang Bi, Fan Qin

**Affiliations:** 1 Qianyuan National Laboratory, Hangzhou 310024, China; renhang_97@163.com; 2 School of Telecommunication Engineering, Xidian University, Xi’an 710071, China; jybi@stu.xidian.edu.cn (J.B.); fqin@xidian.edu.cn (F.Q.)

**Keywords:** beam-scanning, transmitarray antennas, low-cost, wide-angle

## Abstract

In this paper, a multi-feed transmitarray with high-gain, wide-angle beam-scanning, and low-cost features is presented. A novel hybrid phase distribution (HPD) method is proposed to improve the beam-scanning range by combining the single-focal and bifocal principles according to the actual feed illumination area. By using the proposed method, the phase distribution of the transmitarray for different scanning angles can be obtained more accurately, thereby reducing the phase error between the actual and ideal phase distributions. To construct the transmitarray, a three-layer polarization conversion unit cell, consisting of two orthogonal polarizers in the outermost layers and a polarization rotating patch in the middle layer, is designed to provide high-efficiency transmission and full 360° phase coverage. Based on the HPD method, a single-polarized transmitarray antenna with a focal diameter ratio of 0.28 is designed and simulated. The simulated results show that the enhancement of the beam-scanning range is successfully realized. This design can perform a discrete ±60° beam-scanning range with a peak gain of 24 dBi. The gain losses of 0.7 dB at ±30° and 4.7 dB at ±60° are achieved. The cross-polarization levels are about 44 dB and 35 dB at 0° and −60° scanning angles, indicating low cross-polarization of the proposed solution. A five-beam prototype is fabricated and measured for experimental verification purposes. The measured results demonstrate good consistency with the simulations in the main lobe, with slight deviations due to practical fabrication and measurement constraints. The proposed design has advantages such as low-cost, wide beam-scanning angle, high-gain, low-profile and easy fabrication.

## 1. Introduction

The directional beam-scanning antenna is in great demand in many areas such as mobile communications and radar [1,2]. In addition, characteristics such as high-gain, wide-angle, low-complexity, low-cost and easy fabrication increasingly need to be exhibited in the application of beam-scanning antennas. Phased arrays [3,4,5], reflectors [6,7,8] and lenses [9,10,11] are common approaches for realizing high-gain beam-scanning antennas. A phased array antenna is a common solution for wide-angle beam scanning. However, a large number of active modules cause unavoidably high costs and the feed networks increase the design complexity [12]. Reflectors and lenses are both widely used for high-gain beam-scanning applications by adjusting the aperture phase distribution to obtain the desired beams. Compared with reflectors, lenses based on transmitarrays have the advantages of avoiding feed blockage and adding more degrees of freedom to the system [13].

Transmitarray antennas can be made of single or multiple layers of substrates and metallic patches [14]. Generally, a transmitarray antenna consists of many quasi-periodic unit cells in a specific arrangement, each of which has a high transmission magnitude and adequate phase range coverage. The structure of the transmitarray makes it easier to modulate the phase front of the generated beams and obtain the desired characteristics. An easy way to realize a beam-scanning system is to illuminate the transmitarray by a linear feeding array or a moving feed. However, the radiation performance will deteriorate significantly with beam steering because of the serious phase error when the feed deviates from the focal point. Several effective solutions have been proposed to achieve wide-angle beam steering based on a metasurface. In [15], a bifocal method is adopted for the realization of the dielectric transmitarray. The numerical results of the radiation patterns show stable performance in an angular range of ±40°, with low scan loss of 1.2 dB. However, the beam-scanning angle is still limited. In [16], the metasurface lens with elliptical paraboloid phase distribution realizes a scanning coverage of ±60° with a gain drop of 3.7 dB. However, the mentioned design suffered from non-negligible low aperture efficiency. In [17], a 2 bit electronically steerable transmitarray antenna is presented with the beam-scanning capability of ±60° in E- and H-planes and the 3 dB fractional bandwidth is 16.2%. The reconfigurable structure is based on a unit cell with four p-i-n diodes to realize excellent scanning capability, while the cost of active modules and antenna complexity is quite high, which limits its applications in some scenarios. In [18], a transmitarray antenna fed by phased array antennas is proposed to scan across ±50° with a gain loss of 3.3 dB. However, the design of the phased array feed costs a lot and is not convenient to use. A low-cost beam-steering reflectarray with ±70° scan coverage is provided in [19], while the dual-rotation mechanism adds complexity to the applications. As a result, a low-cost easy-fabricated transmitarray antenna that provides a wide-angle beam-scanning capability is of great necessity.

To overcome the inherent problems of beam-scanning antennas, this paper proposes a transmitarray based on a novel hybrid phase distribution (HPD) method and greatly reduces the phase error caused by the mismatch between the actual phase distribution and ideal phase required by different scanning angles. Based on the HPD method, a transmitarray antenna illuminated by a linear feeding array is designed, simulated and measured, which has unique features of wide-angle beam scanning, high-gain, low-cost, low-profile and easy fabrication. This paper is organized as follows. In Section 2, the design of the transmitarray is carefully discussed. Firstly, the operation principle of the HPD is described and the phase error is analyzed mathematically for beams in each desired direction. Then, we introduce the design of the unit cell and the overall transmitarray in detail. The simulation and measured results of the designed transmitarray are given in Section 3. Section 4 gives the conclusion.

## 2. Transmitarray Design Based on HPD Method

In this section, the phase distribution principles of the conventional bifocal design and the proposed hybrid phase distribution (HPD) method are introduced. For clarity, virtual focal points are used to illustrate the phase synthesis process, which should not be confused with the physical feed antennas employed in the fabricated prototype. The differences between the two methods are explained in detail to highlight the advantage of the proposed HPD approach in wide-angle beam scanning.

### 2.1. Operation Principle of the Hybrid Phase Distribution Method

**Figure 1:**
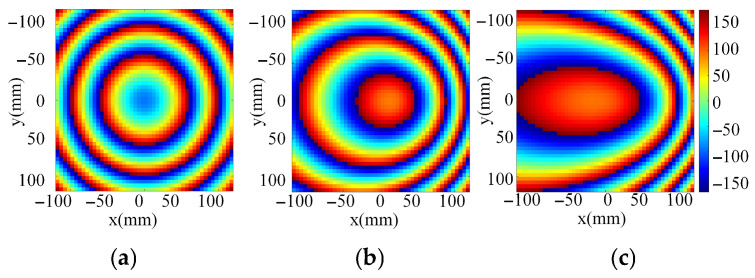
Desired aperture phase distributions at the beam direction of (**a**) 0° with a centered feed, (**b**) −30° with a deviated feed and (**c**) −60° with a deviated feed.

Phase mismatch is the key factor limiting the scanning range of the transmitarray. For example, the desired phase distributions with scanning angles of 0°, −30° and 60° based on a conventional single-focal transmitarray are given in Figure 1. In Figure 1a–c, the aperture diameter is 240 mm × 240 mm and the feeds have offset distances of 0 mm, 51 mm and 92 mm. For a passive transmitarray, the fixed phase distribution is difficult to be adapted to the required phase distributions for different scanning angles. Furthermore, the phase error will increase with the scanning angle, leading to limited scanning angle, gain loss and sidelobe degradation.

To solve above problem, bifocal design method [20,21] was proposed and regarded as an effective approach to realize better scanning performances compared with the conventional single-focal design. However, the bifocal method is only effective for 30° beam scanning and the scanning range is still limited since the phase error increases significantly at an extreme feed offset angle. To further improve the beam-scanning ability, the hybrid phase distribution (HPD) method is presented in this paper, which can significantly reduce the phase error caused by beam scanning and realize an improvement in scanning ability.

**Figure 2:**
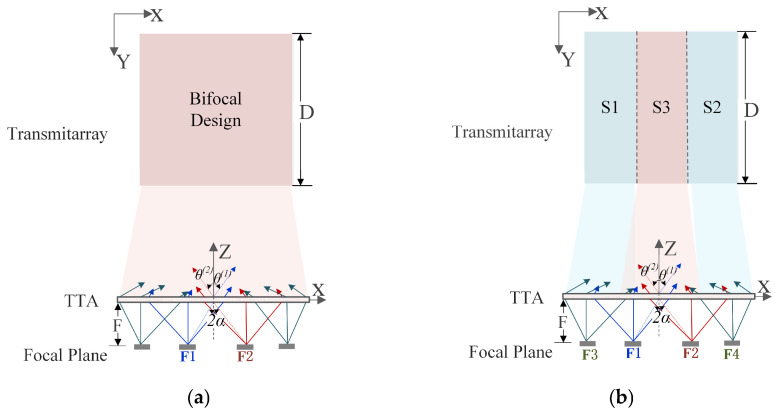
(**a**) Schematic diagram of the bifocal phase aperture design. (**b**) Schematic diagram of the hybrid phase aperture design.

To better understand the proposed HPD method, a typical schematic diagram of the bifocal phase aperture design is depicted in Figure 2a, where F1 and F2 are two virtual focal points with an offset angle of α degrees along the central axis. The phase distribution for the bifocal method is based on two virtual focal points. The required phase distribution based on the bifocal method for the (i,j)-th element can be computed as the mean value of two different phase distributions considering two virtual feeds placed symmetrically in the x-z plane, as shown in the following function.

(1) $$\Phi_{ij}^{bifocal}=(\varphi_{ij}^{\left(1\right)}+\varphi_{ij}^{\left(2\right)})/2$$

where $\varphi_{ij}^{\left(1\right)}$ is the phase distribution when the feed at the position of F1 is turned on, and $\varphi_{ij}^{\left(2\right)}$ presents the phase distribution related to the illumination of the feed at F2. Since the phase distribution for the bifocal method is based on two virtual focal points, the average phase compensation for the two focal points is effective for the feed position around and between the two points. However, the phase mismatch still cannot be ignored when the feed antenna is far away from the virtual focal point, leading to limited scanning angle.

Figure 2b illustrates the proposed HPD method, where F1–F4 are virtual focal points used to derive the aperture phase distribution for different beam directions. These points are introduced only for phase synthesis and are not physical feed antennas. Based on the illuminated areas of different beam states, the transmitarray aperture is divided into three sections, S1, S2, and S3, so that the phase distribution can better match the actual feed illumination and reduce the phase error for wide-angle scanning. It should be noted that Figure 2a involves only two virtual focal points (F1 and F2) corresponding to the bifocal design, whereas Figure 2b introduces four virtual focal points (F1–F4) to enable more flexible phase synthesis in the proposed HPD method. As shown in Figure 2b, the sections S1 and S2 refer to the desired phase distribution for a broadside beam-scanning angle corresponding to feed locations at virtual points F3 (−x1, 0, −F) and F4 (x1, 0, −F), respectively, and S3 is the desired phase distribution based on the bifocal method. All of the design choices for region S3 of the hybrid design and the bifocal design are the same. The main beam of each feed pointing at different angles illuminates different areas on the transmitarray aperture, and S2 receives stronger energy of the incident fields of feeds with a large deviation angle and S3 has stronger excitation with the feed position between F1 and F2. Thus, the phase error at each desired beam direction is expected to be reduced effectively and a larger beam-scanning angle can be realized. In the practical design, the main illumination area of each feed covers more than one section of the transmitarray, and the phase distribution of each section should be carefully adjusted to ensure phase continuity between the adjacent subarrays.

The desired phase distribution for a given scanning angle can be expressed by the following equation:

(2) $$\Phi_{ij}^{desired}=k_{0}\left(R_{ij}-\overset{⃑}{r_{ij}}\times \hat{r_{0}}\right)+∆\varphi$$

where $k_{0}$ is the wavenumber in free space, *R_ij_* denotes to the spatial Euclidean distance between the feed and the (i,j)-th element, ${\vec{r}}_{ij}$ is the position vector of the (i,j)-th element, ${\hat{r}}_{0}$ is the unit vector in the main beam direction, and Δφ is the initial reference phase between the neighboring subarrays, which should be added carefully to ensure phase continuity on the transmitarray aperture.

According to Formulas (1) and (2), the phase distribution based on the HPD method can be obtained by combining the phase distributions of three subarrays, S1, S2 and S3, which can be written as follows:

(3) $$\Phi_{ij}^{hybrid}={\{k}_{0}\left(R_{ij}-\overset{⃑}{r_{ij}}\times \hat{r_{0}}\right)+∆\varphi \}\;\left(\;if\;P\left(i,j\right)\in S1\cup S2\right)\;+\;\left\{(\varphi_{ij}^{\left(1\right)}+\varphi_{ij}^{\left(2\right)})/2\right\}\;(if\;P(i,j)\in S3)\;$$

where P(i, j) denotes to the position of the (i,j)-th element.

To evaluate the initial scanning performance, the phase error between the desired and the designed hybrid aperture phase at different scanning angles can be calculated by using the following equation:

(4) $$E_{ij}=\omega_{ij}\left|\Phi_{ij}^{desired}-\Phi_{ij}^{hybrid}\right|$$

where *ω*_ij_ is the weight factor used to define different contributions of the (*i*, *j*)-th element of the transmitarray to the main beam, which can be obtained by the array theory method [22] or extracting the amplitude of the incident fields on the transmitarray aperture [19].

In practical design, the transmitarray considering the illumination of the real feed antenna is desired. Thus, the real feed source illumination is used instead of ideal magnitude to ensure that the results are closer to the actual situation. More details of the feed antenna can be found in Section 2.3.

**Figure 3:**
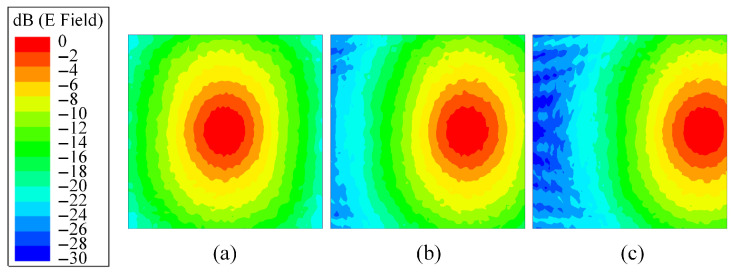
Normalized incident fields on the transmitarray aperture with different feed positions for desired beam direction at (**a**) 0°, (**b**) −30° and (**c**) −60°.

All the electromagnetic simulations presented in this paper, including the unit cell analysis and transmitarray modeling, were performed using ANSYS HFSS (v 2018) (High-Frequency Structure Simulator). The feed fields used in the transmitarray simulations were also obtained from the HFSS. The normalized electric fields of the feed antenna under different feed positions are exported using the HFSS, as shown in Figure 3. Based on the electric field intensity, the factor distribution ωij for the (i,j)-th element can be obtained as the normalized results of the exported values. Of note is that a small focal diameter ratio (F/D) is set as 0.28 to balance the desired phase distribution and the phase error caused by feed deviation. As can be seen, the 10 dB illumination area only occupies part of the transmitarray aperture for each feed position and the illumination levels of these feeds produced on the transmitarray edges are about 20 dB, 10 dB and 3 dB, respectively. Based on the reduced illumination area, the phase distribution on the main illumination area of a deviated feed can be designed more specifically and the phase error for the large-angle scanned beam can be reduced.

**Figure 4:**
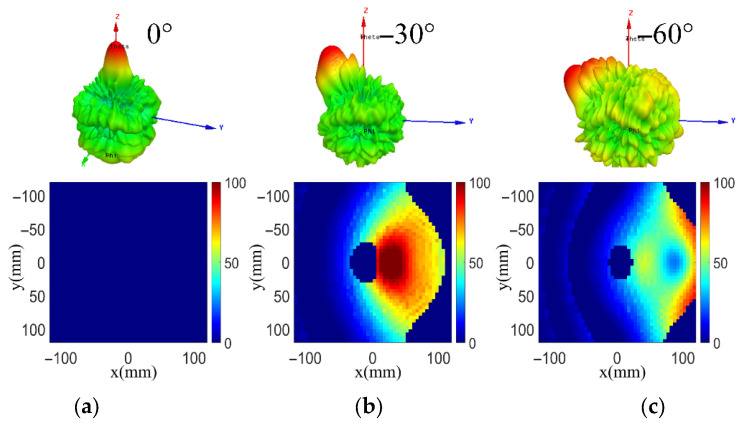
Phase error distributions and radiation patterns of single-focal transmitarray for different beam directions at (**a**) 0°, (**b**) −30° and (**c**) −60°.

The transmitarray based on single-focal design can guarantee beam-scanning at a specific scanning angle, while serious gain loss will appear when the feed deviates far from the focal point. When the feed deviates from the focal point, the phase error caused by the mismatch between the desired phase distribution and the actual phase distribution will increase. As shown in Figure 4, the sidelobe rises as the phase error increases and serious deterioration appears on the radiation patterns of the scanned beams. The phase error for the central beam is 0° and the main beam shows great performance as a focused beam. The phase errors for −30° and −60° scanned beams increase significantly and the corresponding radiation patterns cause deterioration of the high sidelobe which results in the gain drop of the scanned beam.

**Figure 5:**
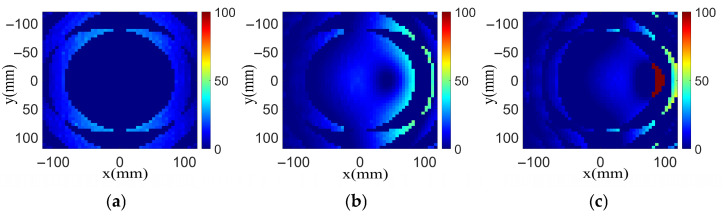
Phase error distributions of bifocal design in consideration of ωij at beam directions of (**a**) 0°, (**b**) −30°, and (**c**) −60°.

**Figure 6:**
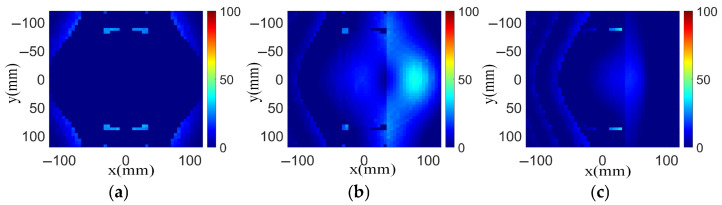
Phase error distributions of hybrid design in consideration of ωij at beam directions of (**a**) 0°, (**b**) −30°, and (**c**) −60°.

The phase error distributions at 0°, −30° and −60° using the bifocal method and HPD method designs are calculated according to Formula (4), as shown in Figure 5 and Figure 6, respectively. The phase error distributions applying the bifocal method are computed with *α* = 33° and *θ* = 29°. The same design choices are employed in both of the bifocal design and section S3 in the hybrid design. The beam directions considered for F3 and F4 are 62° and −62°, respectively. The feeds F3 and F4 have an offset distance x1 of 85 mm. The aperture dimensions are all set as 240 mm in the designs in this section.

**Figure 7:**
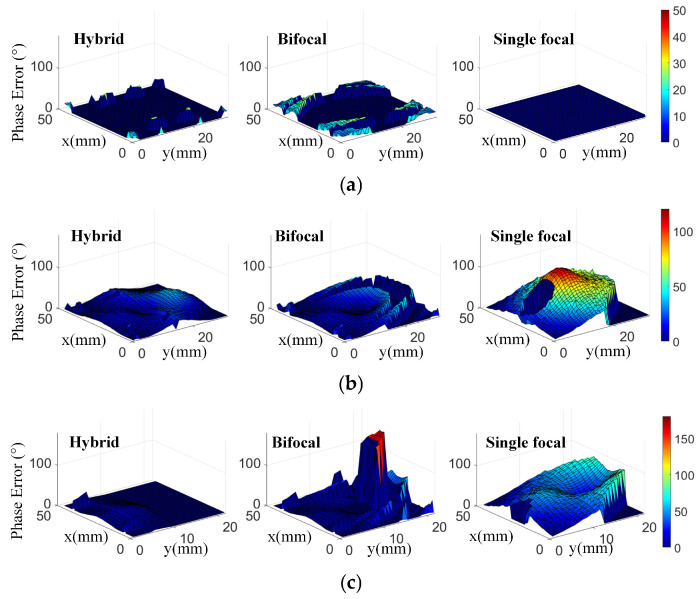
Phase error of hybrid, bifocal and single-focal designs distributed in the main illumination area at beam directions of (**a**) 0°, (**b**) −30° and (**c**) −60°.

To observe the phase error of different methods more visually, we calculated the phase error that is distributed in the main illumination area and plotted the results in 3D view, as shown in Figure 7. These designs have the same feed position at each beam direction. As can be observed, the proposed hybrid transmitarray design reduces the phase error effectively at different scanning angles compared with the bifocal design and classical single-focal design. For the 0° scanning angle, the aperture phase errors using the proposed HPD method are all less than 15°. The phase errors remain less than 30° for the −30° phase distribution. When the scanning beam points at −60°, the phase errors are within 20°. Obviously, optimization for the −30° and −60° phase distributions can be observed for the proposed hybrid design compared with other designs. Conclusively, the transmitarray antenna using the HPD method can reduce the phase error significantly. Based on such a reduced phase error, the aperture phase distribution is optimized and a wider scanning range with stable radiation patterns can be obtained, indicating good scanning performance.

### 2.2. Unit Cell Design

The transmitarray unit cell structure is of great importance in wide-angle beam-scanning transmitarray design to obtain beams in the desired angles. To compensate for the phase delay caused by different feed positions and beam directions, a subwavelength single-polarized unit cell structure is chosen to realize 360° phase coverage and high transmission efficiency with the advantage of broadband in this paper.

**Figure 8:**
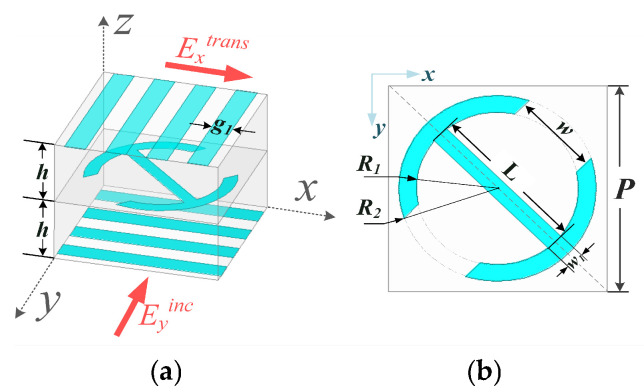
Configuration of the unit cell: (**a**) 3D view; (**b**) metallic pattern in the middle layer (the polarization conversion patch).

The configurations of the employed unit cell are depicted in Figure 8. As shown in Figure 8a, the transmitarray unit cell has a polarization conversion patch in the middle layer sandwiched by two metallic orthogonal grids printed on the dielectric substrate. Assuming that the y-polarized incident wave illuminates the unit cell, the proposed unit cell can convert the y-polarized incident wave into an x-polarized transmission wave, while the x-polarized incident wave is totally reflected. A 2 mm-thick dielectric substrate with a relative permittivity of 2.65 and a dielectric loss tangent of 0.001 is used. Additionally, the total thickness of the transmitarray is 4 mm, corresponding to around 0.13 λ, where λ is the free-space wavelength at the center frequency of 10 GHz. Two metallic grid polarizers are arranged orthogonally with a width of g1 = 0.625 mm. As shown in Figure 8b, the polarization conversion patch with dual ring-shaped resonators has a rotation angle of 45° along the y-axis. The period of the unit cell is set as P = 5 mm, which is around 0.167 λ. The parameters are optimized by the commercial software HFSS using master–slave boundary conditions and the Floquent port. The final optimized parameters are L = 3.7 mm, R1 = 1.85 mm, R2 = 2.26 mm and w1 = 0.4 mm.

**Figure 9:**
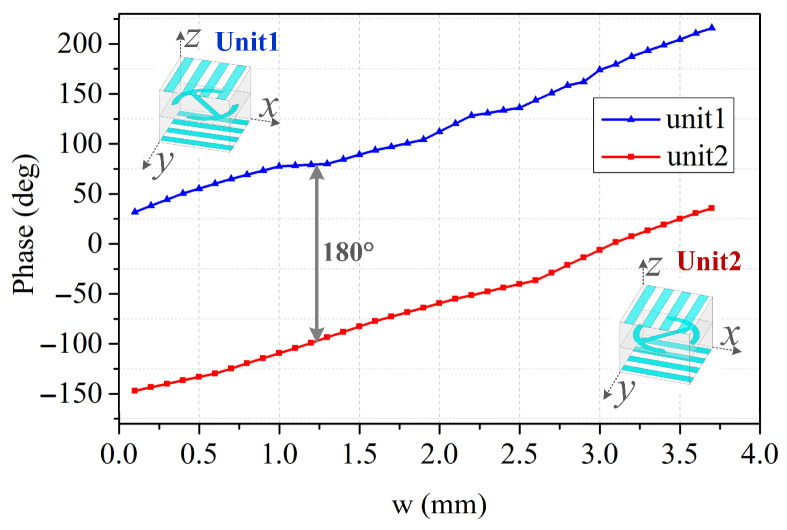
Phase versus “w” of unit1 and unit2 at 10 GHz.

Figure 9 shows the simulated transmission phase versus “w” of the two polarization converters at different frequencies. As the parameter “w” varies from 0.1 mm to 3.7 mm, a phase variation of 186° is provided. By simply rotating the polarization conversion patch of the unit cell by 90°, an additional 186° phase shift can be created [23]. Therefore, an adequate phase range exceeding 360° is successfully obtained. A stable 180° phase difference is provided between two polarization converters, unit1 and unit2, which indicates the broadband characteristics of the transmitarray unit cell [24].

**Figure 10:**
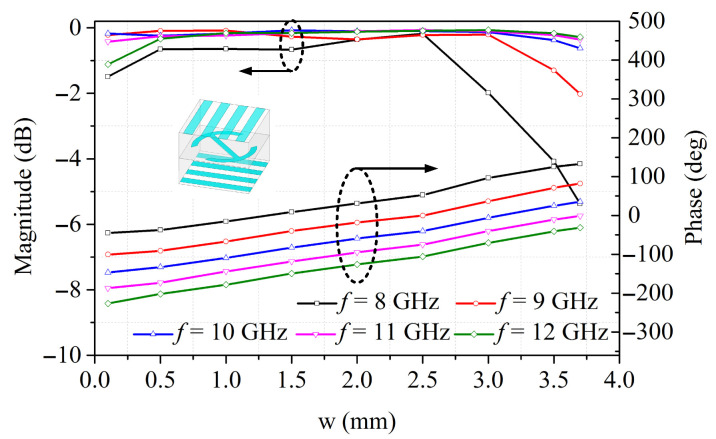
Transmission magnitude and phase versus “w” at different frequencies.

Figure 10 shows the magnitude and phase of transmission coefficient at different frequencies with a normal incident angle. The upper set of curves represents the magnitude, while the lower set corresponds to the phase response. The designed unit cell has a high transmission coefficient better than −0.7 dB at 10 GHz when “w” varies from 0.1 mm to 3.7 mm. Most of the incident energy is transmitted by the unit cell with little loss. In addition, the simulated results demonstrate that the proposed element has a high power conversion ratio close to 1 in a wide frequency band. The transmitarray unit cell is single-polarized and has polarization conversion capability.

**Figure 11:**
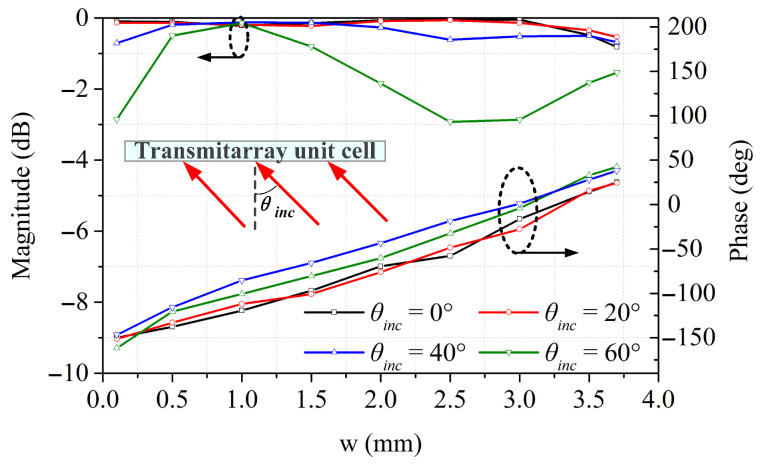
Transmission magnitude and phase versus “w” under different incident angles at 10 GHz.

The incident wave has an oblique angle with the unit cells on the edges of the transmitarray. A unit cell which stabilizes the transmission characteristic as the angle of incidence is varied is needed in the beam-scanning transmitarray system. Figure 11 represents the magnitude and phase of the transmission coefficients under different incident angles at 10 GHz. The magnitude is higher than −1 dB for incident angles up to 40°, and is higher than −3 dB even at an incident angle of 60°. The transmission phase variation remains smaller than 30° at extreme incident angles. Hence, the proposed unit cell shows modest sensitivity of incident angles and can be a promising candidate to design a wide-angle beam-scanning transmitarray antenna.

### 2.3. Wide-Angle Beam-Scanning Transmitarray

**Figure 12:**
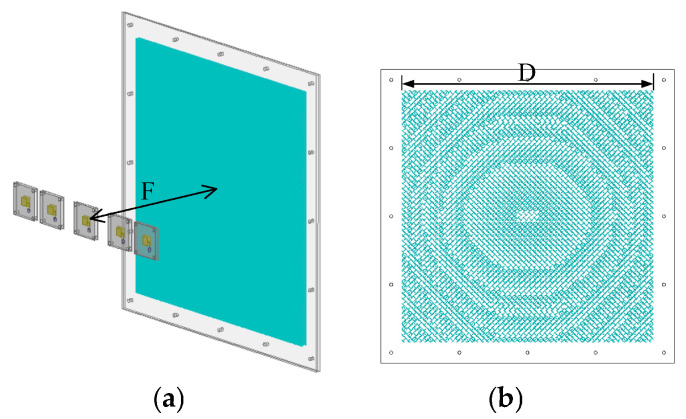
Structure of the proposed transmitarray antenna. (**a**) A 3D view with the linear feeding array. (**b**) Topology structure of middle layer of the proposed transmitarray.

The unit cell has been adopted to design a square transmitarray with 48 × 48 elements, corresponding to a size of D = 8 and λ = 240 mm. The distance between the focal plane and the transmitarray is 67.2 mm (2.24λ), with an F/D of 0.28. The three-dimensional (3D) view and the topology structure of the middle layer of the simulated transmitarray are shown in Figure 12. Five feeds are placed in specific positions and when the five feed elements illuminate the transmitarray separately, the main beams in corresponding transmission directions are generated. Since the transmitarray unit cell is single-polarized, the proposed solution is single polarization and has the capability of polarization conversion. Furthermore, the proposed HPD method can also be applied to transmitarray designs with other polarization modes such as circular polarization and dual polarization. Thus, the proposed method is also suitable for beam-scanning systems in other application scenarios such as satellite and radar communications.

**Figure 13:**
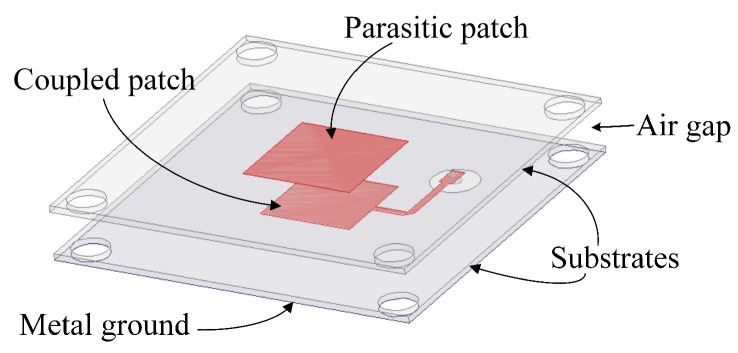
A 3D view of the feed antenna.

**Figure 14:**
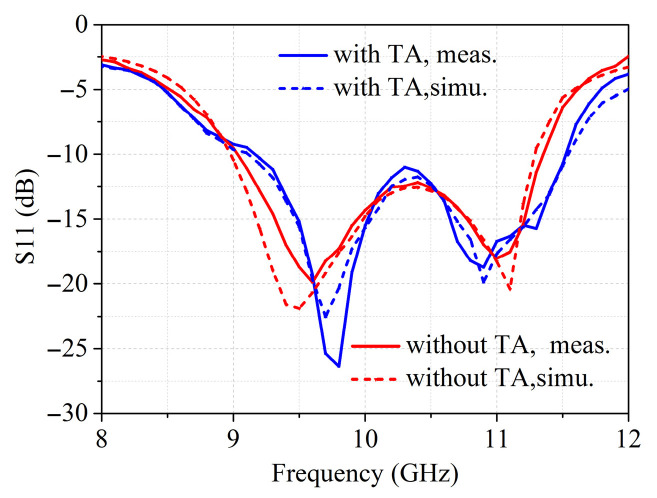
Simulated and measured S11 with and without loading TA versus frequency.

**Figure 15:**
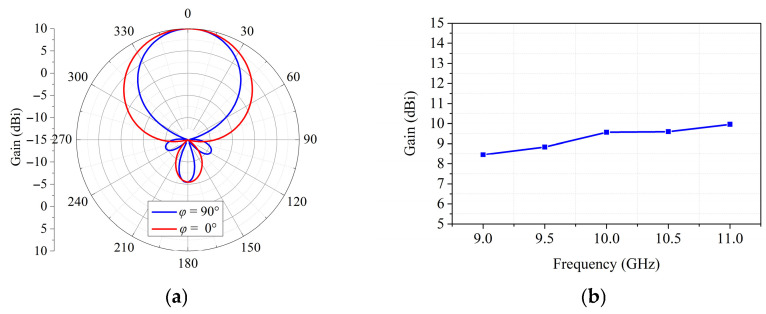
Characteristics of the feed: (**a**) 2D radiation pattern; (**b**) the curve of gain versus frequency.

Figure 13 gives the 3D view of the feed antenna, which consists of two 0.5 mm-thick substrates of RO4350B, metal ground, a 7.5 × 7.5 mm^2^ coupled patch, a 9 × 9 mm^2^ parasitic patch and an air gap of 2.6 mm between the two layers. The feed antenna has a gain of 9.8 dBi. As can be seen in Figure 14, the simulated and measured S11 of the feed antenna are less than −10 dB from 9 GHz to 11 GHz and the loading of the transmitarray has little effect on the characteristics of the feed antenna. The working frequency bandwidth is wide enough to meet the excitation demand of the proposed transmitarray and to verify the design. The 2D radiation pattern of the feed at 10 GHz is shown in Figure 15a; the radiation patterns of the feed have good symmetry in the E- and H-planes. The curve of gain versus frequency is plotted in Figure 15b which shows flat gain variation in the frequency range from 9 GHz to 11 GHz.

**Figure 16:**
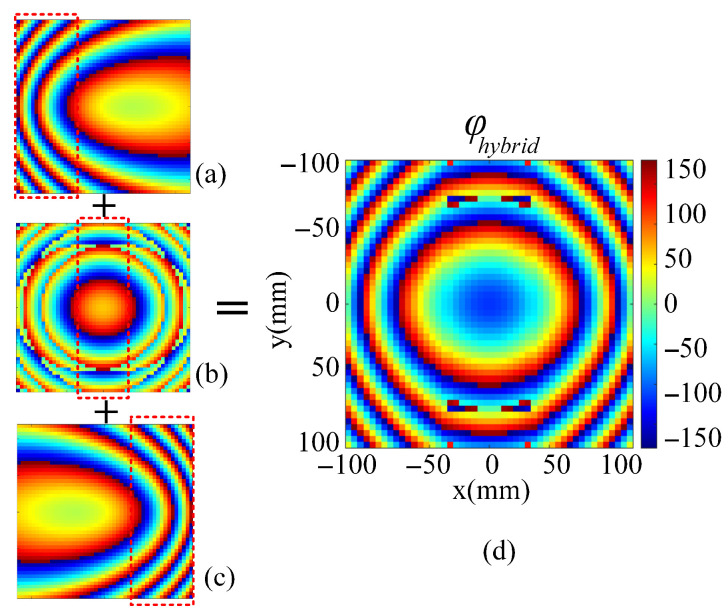
Phase distribution based on hybrid phase distribution design. (**a**) Desired phase distribution for 60° scanning beam. (**b**) Bifocal phase distribution. (**c**) Desired phase distribution for −60° scanning beam. (**d**) Hybrid phase distribution.

Based on the analysis above, the required hybrid phase distribution of the transmitarray can be obtained. Figure 16a–c give the phase distributions calculated with different formulas. Figure 16a is the desired phase distribution at a scanning angle of 60° with the feed at F3 (−85 mm, 0, −F), Figure 16b shows the bifocal phase distribution with α = 33° and θ = 29°, and Figure 16c is the desired phase distribution at a scanning angle of −60° with the feed at F4 (85 mm, 0, −F). To minimize the phase error in the desired beam direction, the size ratio of the three parts is carefully set as 17:14:17 and the hybrid phase distribution is plotted in Figure 16d.

## 3. Results and Discussion

**Figure 17:**
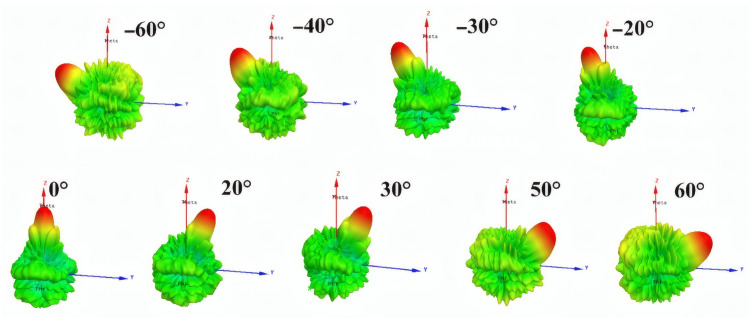
Simulated radiation patterns at 10 GHz pointing at −60°, −40°, −30°, −20°, 0°, 20°, 30°, 50° and 60°.

**Figure 18:**
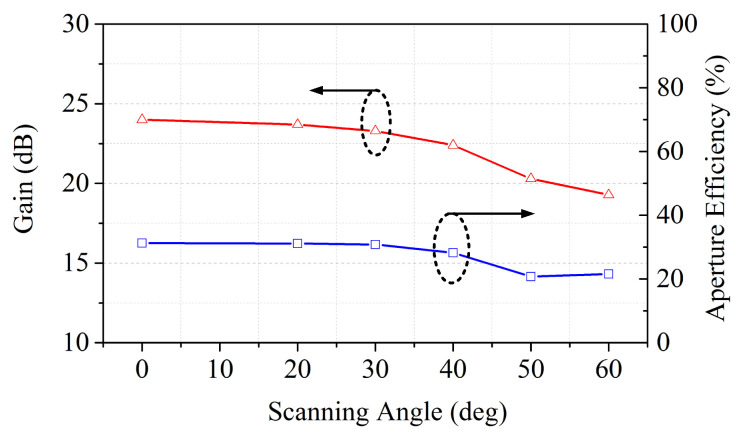
Simulated gain and aperture efficiency as the gain over the projected aperture at 10 GHz versus scanning angle.

Based on the above discussion, a beam-scanning transmitarray based on hybrid phase distribution is simulated. By exciting each feed antenna located at different positions separately, main beams with scanning angles of −60°, −40°, −30°, −20°, 0°, 20°, 30°, 50° and 60° can be generated. The simulated radiation patterns at 10 GHz pointing at different beam directions are shown in Figure 17. The sidelobe level increases as the scanning angle increases from 0° to 60° and the scanning beams present a gain loss of 4.7 dB. Figure 18 shows the simulated gain and the corresponding aperture efficiency versus scanning angle curves. The projected aperture dimension is used to calculate the aperture efficiency for scanned beams, which can be written as A*cos θ (A is the aperture dimension; θ is the scanning angle). The gains of the transmitarray antenna under scanning angles of 0°, 20°, 30°, 40°, 50° and 60° are 24.0 dBi, 23.7 dBi, 23.3 dBi, 22.4 dBi, 20.3 dBi and 19.3 dBi, respectively. Thus, the aperture efficiency, which is defined as the gain over the projected aperture, can be calculated as 31.2%, 31.1%, 30.7%, 28.2%, 20.7% and 21%, accordingly. In this paper, the aperture efficiency is calculated based on the full aperture calculation. However, the illumination area produced by each feed only occupies part of the whole transmitarray aperture, which leads to low aperture efficiency. For the centered feed, the aperture efficiency is about 54.6% for a 0° scanned beam based on an effective aperture calculation in the proposed design.

**Figure 19:**
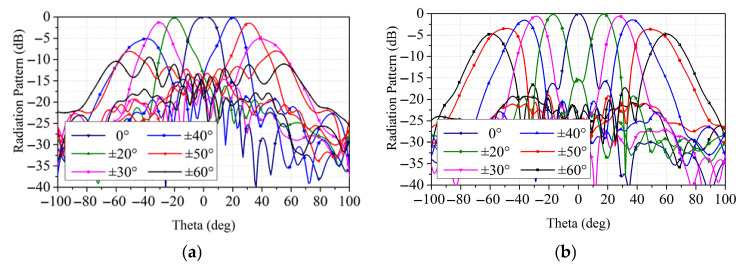
(**a**) Simulated radiation patterns at 10 GHz with different scanning angles of the bifocal design. (**b**) Simulated radiation patterns at 10 GHz with different scanning angles of the hybrid design.

To further demonstrate the performance improvement in the proposed hybrid employment, Figure 19 gives the simulated radiation patterns of the bifocal design and the proposed hybrid design under different beam directions. A detailed comparison between the two designs is given in Table 1. The proposed hybrid design has a higher gain of 24 dBi in the 0° beam direction compared with the bifocal transmitarray. As can be seen in Figure 19a, the bifocal transmitarray can obtain stable radiation performance in a scanning range of ±40° and the main beam has serious deterioration at ±50° and ±60° scanning angles at 10 GHz. The proposed hybrid design achieves a wider scanning range of ±60° with stable radiation patterns, and the scan loss is less than 4.7 dB, as shown in Figure 19b. Additionally, the bifocal transmitarray can only reach a scanning angle of ±30° with a gain loss of 1.4 dB, while the hybrid design has a gain loss of 0.7 dB in a scanning range of ±30°. Compared with the bifocal transmitarray, the proposed design realizes a wider scanning range with lower scan loss and can be a possible candidate for a wide-angle beam-scanning prototype.

**Figure 20:**
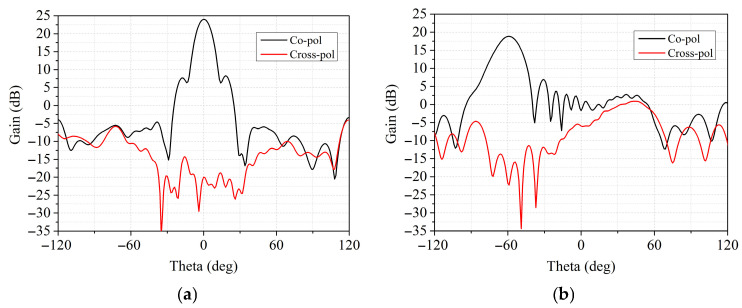
Simulated results of co-polar and cross-polar at 10 GHz at beam-scanning angles of (**a**) 0° and (**b**) 60°.

The proposed transmitarray has the capability of polarization conversion. The simulated co-polar and cross-polar at beam-scanning angles of 0° and −60° are given in Figure 20. As can be seen, the cross-polarization levels are about 44 dB and 35 dB at 0° and −60° scanning angles, which indicates that the proposed antenna has low cross-polarization.

**Figure 21:**
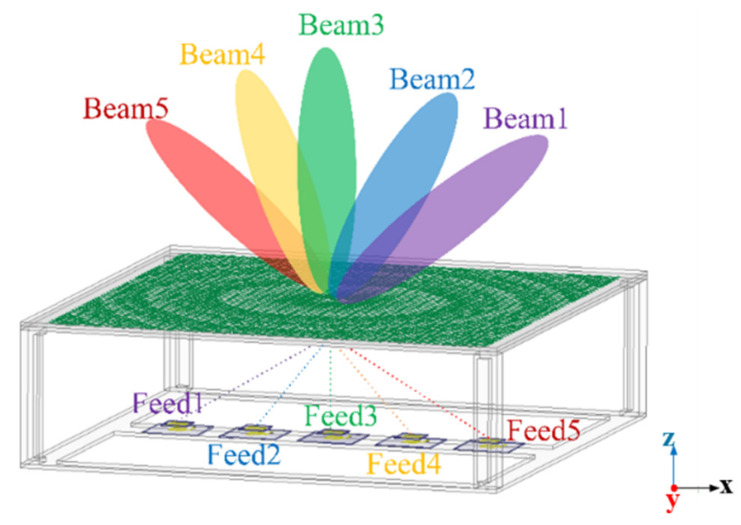
Schematic diagram of the proposed transmitarray antenna.

**Figure 22:**
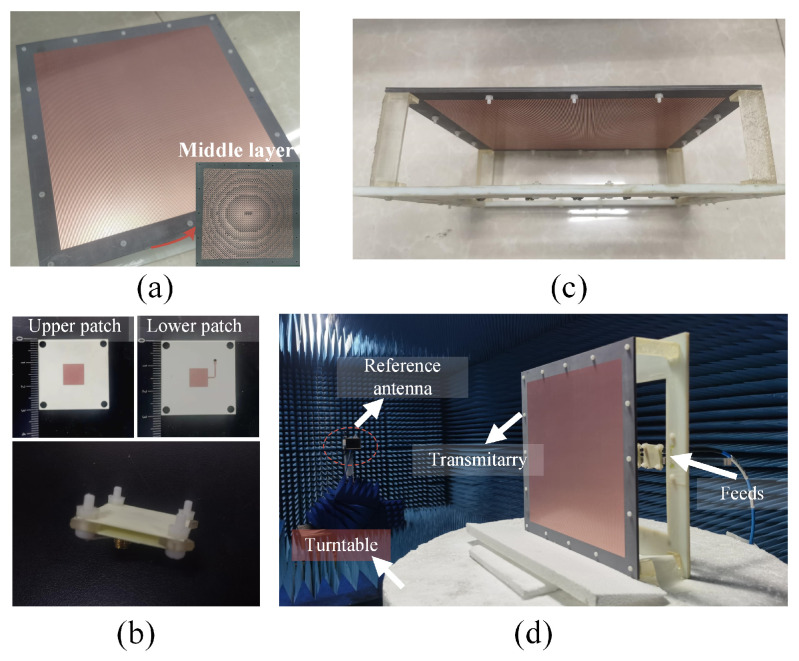
(**a**) Designed transmitarray. (**b**) Feed antenna. (**c**) Transmitarray with feeding array. (**d**) Fabricated transmitarray antenna in anechoic chamber.

To validate the effectiveness of the proposed wide-angle beam-scanning mechanism, the designed transmitarray antenna with a feeding array of five physical feed antennas is fabricated and measured. The schematic diagram of the proposed transmitarray antenna is shown in Figure 21. Five pencil beams are successfully generated with five feeds illuminated the transmitarray separately. For example, Beam1 refers to the scanned beam with Feed1 excited and Beam2 refers to the scanned beam with Feed2 excited, etc. The overall size of the transmitarray is 280 × 280 mm^2^. Five feed antennas are placed in order with x-coordinates of −92 mm, −51 mm, 0 mm, 51 mm, and 92 mm. Four plastic supports produced by a 3D printer are utilized to build an air space between the transmitarray and the feeding source. The plastic supports are considered in the simulation as well. The transmitarray based on the hybrid phase distribution method is developed, as shown in Figure 22a, together with the linear feeding array in Figure 22b,c, which shows the structure of the feed antenna, and the fabricated transmitarray prototype is measured in an anechoic chamber, as presented in Figure 22d.

**Figure 23:**
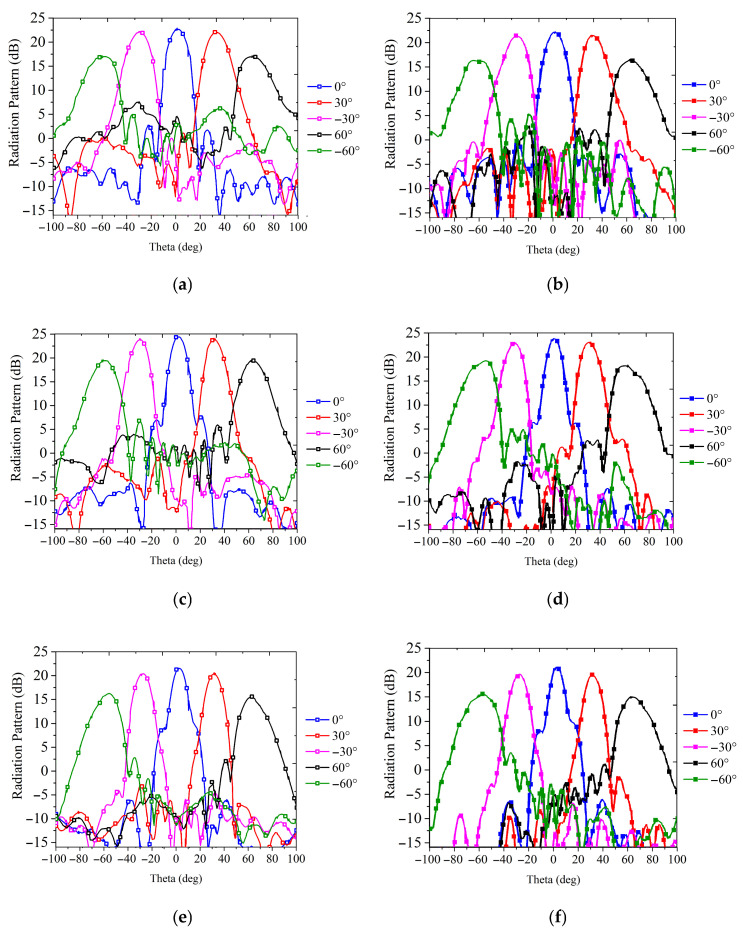
Simulated and measured radiation patterns at 9, 10, and 11 GHz. (**a**) Simulated and (**b**) measured results of 9 GHz, (**c**) simulated and (**d**) measured results of 10 GHz, (**e**) simulated and (**f**) measured results of 11 GHz.

**Figure 24:**
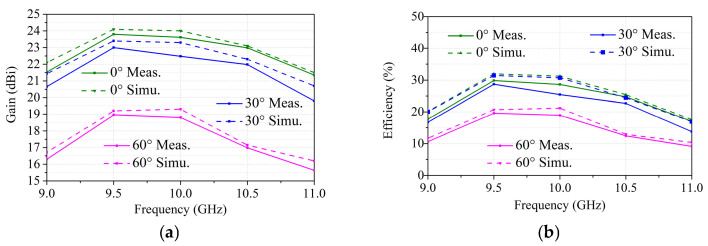
(**a**) Gain versus frequency at 0°, 30° and 60° scanning angles. (**b**) Aperture efficiency versus frequency at 0°, 30° and 60° scanning angles.

The simulated and measured radiation patterns of the transmitarray fed by a feeding array at specific scanning angles of 0°, ±30° and ±60° and different frequencies are plotted in Figure 23. To ensure a clear and consistent visualization of the scanning beams, all data plots have been separated into individual subplots for simulation and measurement. As observed from the patterns, the main lobe characteristics, including beam pointing accuracy and peak gain, demonstrate good consistency between the simulated and measured results. At the X band, the beam direction remains stable at different frequencies. The maximum measured gain is 23.6 dBi at 10 GHz, with a gain loss of 4.8 dB. The gain and aperture efficiency versus frequency at 0°, 30°and 60° scanning angles are plotted in Figure 24. The measured 1 dB gain bandwidths are about 14.6% and 18% at angles up to 0° and 60°, respectively. The measured 3 dB gain bandwidths are about 25% and 24.5% at angles up to 0° and 60°, respectively.

However, slight deviations between the simulation and measurements are present in Figure 23 and Figure 24, particularly at wide scanning angles such as 60°. These discrepancies primarily result from two practical factors. First, fabrication tolerances and minor physical deviations during the manufacturing and assembly of the multi-layer transmitarray introduce slight phase errors across the aperture. As the beam is steered to wider angles using the hybrid phase distribution method, the radiation pattern becomes highly sensitive to these phase errors, leading to degraded SLLs compared to ideal simulation conditions. Second, measurement environment scattering plays a role. At wide scanning angles, the radiation is more susceptible to unavoidable scattering and diffraction from the antenna prototype supports and the feeding structures in the anechoic chamber, which are not completely mirrored in the simulation model.

Moreover, a comparison of the performances of different beam-scanning antennas is listed in Table 2. Compared with Ref. [16], our design exhibits higher peak gain with smaller aperture size in a scanning range of ±60°. The beam steering of the proposed transmitarray does not need active modules, which reduces both of the cost and complexity compared with the phased array antenna solutions in Ref. [18]. This paper also provides more convenience to realize beam-scanning performance in practical use compared with Ref. [19], which needs dual rotation of the feed and reflectarray. It can be observed that the proposed transmitarray design exhibits a wider scanning range with less gain loss. The total profile of the proposed transmitarray is only 2.4λ, which is much lower than other beam-scanning antennas. The single-polarized structure can be applied to base stations for mobile communications. Moreover, the proposed HPD method is also suitable for other polarization designs such as circular polarization and dual polarization. By applying the proposed hybrid phase distribution method to other polarization designs, the beam-scanning system can also have extended application in satellite and radar communications. Therefore, the designed transmitarray antenna has the advantages of low-cost, low-profile and easy fabrication, and is expected to be a possible candidate as a wide-angle beam-scanning prototype to fulfill practical demands.

## 4. Conclusions

This paper demonstrates a novel hybrid phase distribution method for low-cost high-gain beam-scanning transmitarray antenna design at the X band. A single-polarized transmitarray with wide-angle beam-scanning capability is designed and validated. The proposed HPD method jointly utilizes the bifocal and single-focal phase distributions under the consideration of the feed illumination and relative positions, which greatly improves the accuracy of phase distribution at different scanning angles. A prototype with a linear feeding array based on the proposed method is simulated, fabricated and measured. The proposed HPD method achieves an improved beam-scanning range by reducing the phase error, compared with conventional single-focal and bifocal designs. The prototype can generate five discrete beam states covering an angular range of approximately ±60°. The peak gain is 24 dBi, with a gain loss of 0.7 dB at ±30° and 4.7 dB at ±60°. Meanwhile, a low-profile property is obtained by the proposed design, which is only 72.2 mm (2.4λ at 10 GHz). The experimental results indicate that while the main lobe characteristics demonstrate good consistency with the simulations, slight performance deviations are present due to unavoidable fabrication tolerances and measurement environment scattering. Overall, the proposed HPD method effectively improves the wide-angle beam-scanning performance while maintaining the advantages of being low-cost and low-profile, showing great potential for future wireless communication systems. Such appealing features and excellent performances mean that it has great potential for applications in base stations for mobile communication. Furthermore, the hybrid phase distribution method also can be applied to other polarization designs, bringing extended applications for beam-scanning systems in satellite and radar communication.

## Figures and Tables

**Table 1:** Comparison between bifocal design and hybrid design.

	Bifocal Design	Hybrid Design
Peak gain	21.75 dBi	24 dBi
Efficiency	18.6%	31.2%
Scanning range	±40°	±60°
Gain loss/scanning angle	5.1 dB/±40° 1.4 dB/±30°	4.7 dB/±60° 0.7 dB/±30°

**Table 2:** Comparison of designed beam-scanning antennas.

Ref.	Mechanism	Center Frequency	F/D	Plane Size	Profile	Peak Gain	Polarization	Gain Loss/Scanning Range
[15]	Feed Movement	30 GHz	1	15.6λ × 15.6λ	15.6λ	27.37 dBi	LP	1.2 dB/±40°
[16]	Feed Movement	30 GHz	0.2	10.5λ × 10.5λ	2.3λ	18.5 dBi	LP	3.7 dB/±60°
[17]	Electronic Steering	29 GHz	0.67	7λ × 7λ	4.7λ	19.8 dBi	LP	5 dB/±60°
[18]	Phased Array	39 GHz	0.28	16λ × 16λ	4.5λ	31.1 dBi	LP	3.3 dB/±50°
[19]	Dual Rotation	12 GHz	1	π × 5λ × 5λ	10λ	26.23 dBi	LP	3.1dB/±60° 4.9 dB/±70°
This Work	Multi Feeds	10 GHz	0.28	8λ × 8λ	2.4λ	24 dBi	LP	0.7 dB/±30° 4.7 dB/±60°

λ is the free-space wavelength at the center frequency of the corresponding antenna.

## Data Availability

Data are contained within the article.
